# Exercise Attenuates Neuroinflammation in Chronic Restraint Stress Induced Depression Model by Downregulating GDF15 Expression

**DOI:** 10.1155/jimr/6816624

**Published:** 2026-04-09

**Authors:** Junrui Chen, Chunxiao Du, Jiawen Lu, Meng Liang, Miaonan Sun, Yanmin Lyu, Mengying Huang, Jixiang Sun, Yuxiang Li, Zhiding Wang, Ge Li, Gencheng Han

**Affiliations:** ^1^ Beijing Institute of Basic Medical Sciences, Beijing, China, bjmu.edu.cn; ^2^ Joint National Laboratory for Antibody Drug Engineering, Key Laboratory of Cell and Molecular Immunology, School of Medical Sciences, Henan University, Kaifeng, China, henu.edu.cn; ^3^ Department of Anesthesiology, The First Medical Center of the PLA General Hospital, Beijing, China; ^4^ Department of Rheumatology and Immunology, The First Hospital of Jilin University, Changchun, China, jlu.edu.cn; ^5^ School of Basic Medical Sciences, Anhui Medical University, Hefei, China, ahmu.edu.cn

**Keywords:** chronic restraint stress, exercise, neuroimmune inflammation

## Abstract

Exercise has been shown to alleviate depressive symptoms and enhance bodily functions. However, the precise mechanisms underlying its antidepressant effects remain incompletely understood. In this study, we observed that treadmill exercise may exert antidepressant effects in a chronic restraint stress (CRS) mouse model by modulating the proinflammatory Growth Differentiation Factor 15 (GDF15)‐extracellular signal‐regulated kinase (ERK) signaling pathway. Treadmill exercise improved cognitive behaviors in CRS mice and alleviated neuroinflammation, as evidenced by decreased expression of TNF‐α, IL‐1β, IL‐6, and attenuated microglia activation. Intriguingly, we found that treadmill exercise inhibited the expression of GDF15, a biomarker associated with many immune disorders, which is increased following CRS. Mechanistically, treadmill exercise may attenuate neuroinflammation by suppressing GDF15‐induced ERK activation. We thus identified a novel mechanism by which treadmill exercise attenuates depression. Modulation of the GDF15‐ERK pathway may have therapeutic implications for depression.

## 1. Introduction

Per the Global Burden of Disease Project [1], depression is currently the leading cause of disability among nonfatal neuropsychiatric disorders and is projected to rank among the top three causes of global disease burden by 2030. Presently, pharmacotherapy remains the primary treatment for depression. However, its efficacy is often limited by side effects, the potential for addiction, high cost, and poor patient compliance. Consequently, drug treatment outcomes are often unsatisfactory, and patients’ quality of life remains seriously impaired. Recently, physical activity has been shown to be associated with decreased symptoms of depression and anxiety. Exercise can not only enhance physical health and reduce disease risk but also improve mental well‐being. Reflecting this, clinical practice guidelines in the United States, United Kingdom, and Australia now recommend physical activity as a core component of depression treatment [2]. For example, Salgureo et al. [3] found a significant inverse correlation between physical activity levels and Geriatric Depression Scale (GDS) scores in a study of 436 elderly Spanish individuals (aged 60–98 years), where greater physical activity was associated with lower depression severity. However, the systematic and comprehensive mechanism by which exercise alters depressive behaviors has not been fully elucidated.

Neuroimmune dysregulation plays a key role in the pathogenesis of depression [4]. An acute inflammatory state, characterized by elevated proinflammatory cytokines, is associated with acute emotional distress [5]. In patients with major depressive disorder (MDD), levels of the proinflammatory cytokines IL‐6, TNF‐α, and IL‐1β are increased, whereas levels of TGF‐β and IFN‐γ are reduced [6, 7]. Microglia, the resident macrophages of the central nervous system, act as the first line of immune defense in the brain. Excessive activation of microglia, which can be triggered by pathways such as extracellular signal‐regulated kinase (ERK) or NF‐κB, has been reported to contribute to the pathogenesis of depression [8–10]. In the LPS‐induced depression model, microglia are activated and contribute to neuroinflammation by secreting proinflammatory cytokines [11]. Consequently, novel treatment strategies for depression that target neuroinflammation and its underlying mechanisms are now widely investigated.

Growth differentiation factor 15 (GDF15), a member of the transforming growth factor β superfamily, is a myokine expressed in and secreted by various organs and tissues. Its expression is induced in diverse physiological and pathophysiological states. GDF15 is involved in the physiopathology of numerous conditions, including obesity, cancer, and depression, and is also associated with the aging process [12, 13]. Overproduction of GDF15 has been reported to promote inflammation and exacerbate diseases such as human rhinovirus (HRV) infection and pneumonia [14]. Intriguingly, several studies report that both aerobic exercise and resistance exercise decrease circulating GDF15 levels in humans [15–17]. It is therefore of great interest to determine whether the modulation of GDF15 by exercise plays a role in the neuroimmune disorders underlying depression.

ERK, a central component of the mitogen‐activated protein kinase (MAPK) pathway, plays a well‐established role in neuronal plasticity, survival, and the therapeutic action of conventional antidepressants [18–20]. Notably, aberrant elevation of phosphorylated ERK (p‐ERK) in the brain under chronic stress conditions strongly correlates with depressive‐like behaviors [21]. This pathological hyperactivation of ERK is increasingly recognized as a contributor to maladaptive neurobiological processes. In particular, sustained ERK signaling within microglia and other glial cells is a well‐documented driver of proinflammatory cytokine production, thereby amplifying neuroinflammation, which is a core pathological feature of depression [22–25]. However, the mechanisms regulating ERK during neuroinflammation and whether interventions such as exercise exert antidepressant effects by modulating ERK activation remain to be determined.

To further elucidate the antidepressant effects of exercise, we employed the chronic restraint stress (CRS) mouse model to investigate the underlying mechanisms, with the aim of providing evidence for the use of exercise in the prevention and treatment of depression. We found that treadmill exercise counteracted the CRS‐induced elevation of GDF15 and the concomitant activation of ERK. These findings suggest that treadmill exercise may improve cognition, at least in part, by inhibiting GDF15‐mediated neuroinflammation.

## 2. Results

### 2.1. Treadmill Exercise Alleviates Depressive‐Like Behaviors in CRS Mice

The CRS mouse model was established first to investigate the antidepressant effects of exercise. Based on established protocols [26–30] and in accordance with the consistency of the mice exercise parameters, we designed the following experimental timeline (Figure 1A). The control group (Ctrl group) did not receive any treatment. The CRS group was restrained daily for 6 h over 14 consecutive days using a custom‐built apparatus. The CRS with treadmill exercise (CT group) underwent the same restraint protocol, followed by daily treadmill exercise at 12 m/min for 1 h. Upon completion of the experimental modeling, the elevated plus maze (EPM), tail suspension test (TST), and forced swimming test (FST) were administered. Subsequently, tissue and plasma samples were collected for further analysis. Behavioral test results demonstrated that CRS successfully induced depression‐like behaviors, as evidenced by an increase in immobility time in the TST compared to the Ctrl group. Conversely, the CT group exhibited significantly reduced immobility time compared to the CRS group (Figure 1B, one‐way ANOVA followed by Dunnett’s post hoc test, *p*  < 0.0001, *F* [2, 21] = 25.60). Similarly, in the FST, the CRS group displayed prolonged immobility relative to both the Ctrl and CT groups (Figure 1C, one‐way ANOVA followed by Dunnett’s post hoc test, *p*  < 0.0001, *F* [2, 21] = 69.60). In the EPM, no differences in general locomotor ability were observed among groups (Figure 1D, one‐way ANOVA followed by Dunnett’s post hoc test, *p* = 0.1112, *F* [2, 21] = 1.676), indicating that subsequent anxiety‐related measures were not confounded by mobility. Compared to the Ctrl group, the CRS group exhibited a significant reduction in the total distance moved in (Figure 1E, one‐way ANOVA followed by Dunnett’s post hoc test, *p* = 0.0005, *F* [2, 21] = 11.26), frequency of entries into (Figure 1F, one‐way ANOVA followed by Dunnett’s post hoc test, *p* = 0.0025, *F* [2, 21] = 8.109), and time spent in the open arms (Figure 1G, one‐way ANOVA followed by Dunnett’s post hoc test, *p* = 0.0012, *F* [2, 21] = 9.472), while the latency to first enter the open arm (Figure 1H, Kruskal–Wallis test followed by Dunn’s post hoc test, *p* = 0.0003, Kruskal–Wallis statistical magnitude = 16.56) was significantly increased. In contrast, the CT group did not show significant differences from the Ctrl group on any of these parameters (Figure 1E–H). Representative movement trackplots and heatmaps for the EPM are presented in Figure 1I. Since dysregulation of the hypothalamic–pituitary–adrenal (HPA) axis is a hallmark of depression, we also measured plasma corticosterone levels at the end of the experiment. The result showed that treadmill exercise decreased plasma corticosterone level in CRS mice (Figure 1J, Welch’s ANOVA followed by Dunnett’s T3 post hoc test, *p*  < 0.0001, *W* [2.000, 12.81] = 14.78). Together, these findings suggest that treadmill exercise mitigates depressive‐like behaviors and normalizes HPA axis dysfunction in CRS mice.

Figure 1Treadmill exercise ameliorates depression‐like behaviors and HPA axis dysregulation in CRS mice. (A) Experimental timeline (created in BioRender, https://BioRender.com/iyvrkua); (B) TST immobility time was analyzed; (C) FST immobility time was analyzed; (D) EPM average velocity was analyzed; (E) EPM distance in open arms was analyzed; (F) EPM open arm entries were analyzed; (G) EPM time in open arms was analyzed; (H) EPM latency to first open arm entry was analyzed; (I) representative movement traces (up) and heatmaps (down) in EPM; (J) plasma corticosterone levels (ng/mL) were analyzed by enzyme‐linked immunosorbent assay (ELISA) after the behavior tests. Each experiment was independently repeated three times. *n* = 8/group,  ^∗^ *p*  < 0.05,  ^∗∗^ *p*  < 0.01,  ^∗∗∗^ *p*  < 0.001,  ^∗∗∗∗^ *p*  < 0.0001, and ns, no significant difference. One‐way ANOVA (B, C, D, E, F, G) followed by Dunnett’s post hoc test. Welch’s ANOVA (J) followed by Dunnett’s T3 post hoc test. Kruskal–Wallis test (H) followed by Dunn’s post hoc test.

**Figure:**
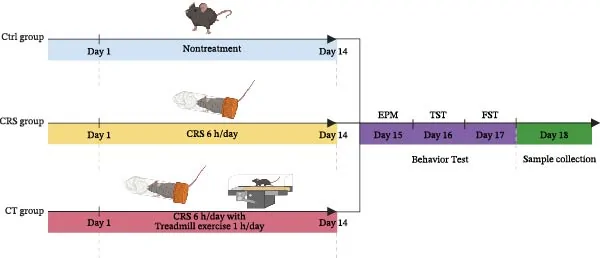
(A)

**Figure:**
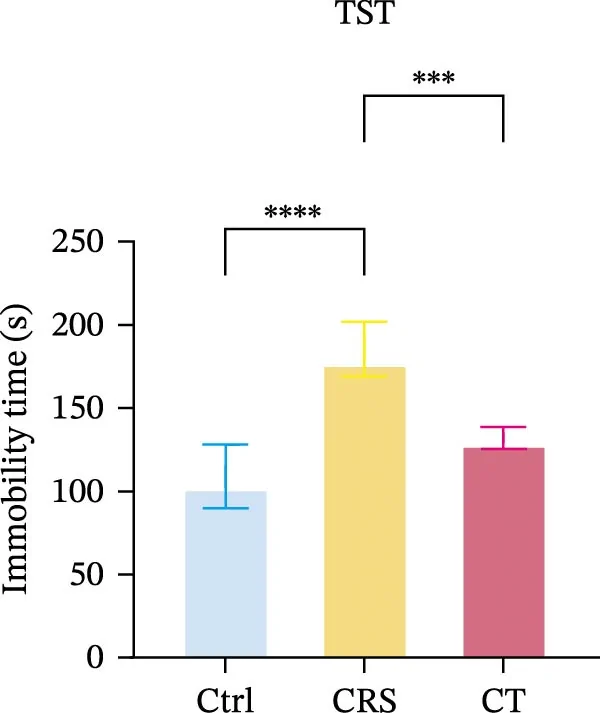
(B)

**Figure:**
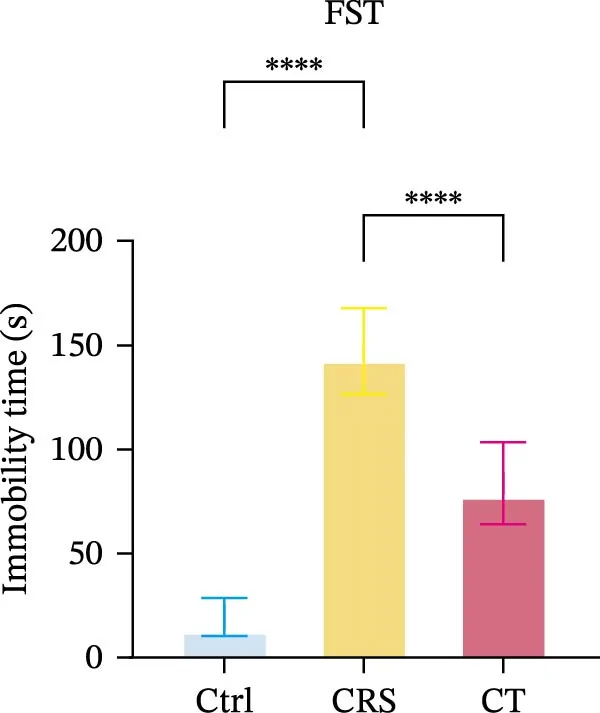
(C)

**Figure:**
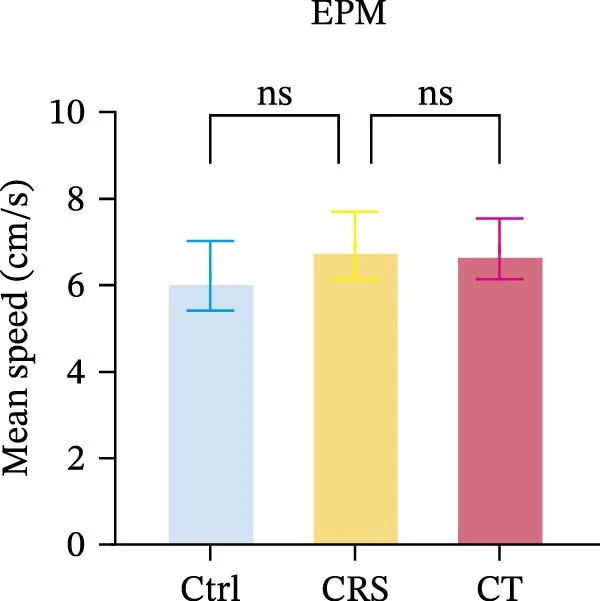
(D)

**Figure:**
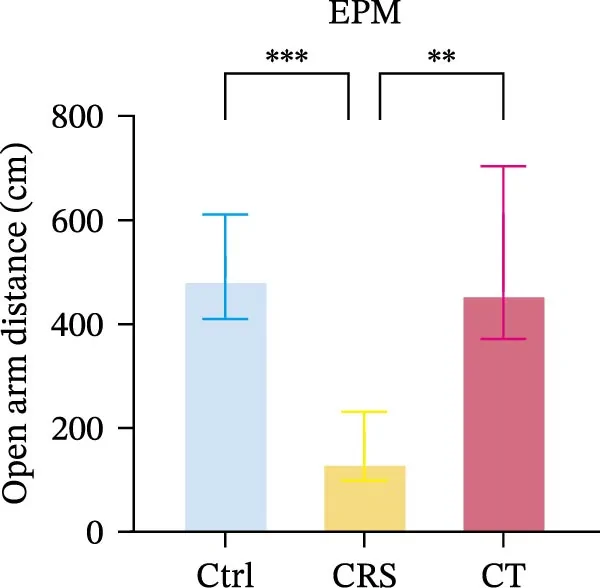
(E)

**Figure:**
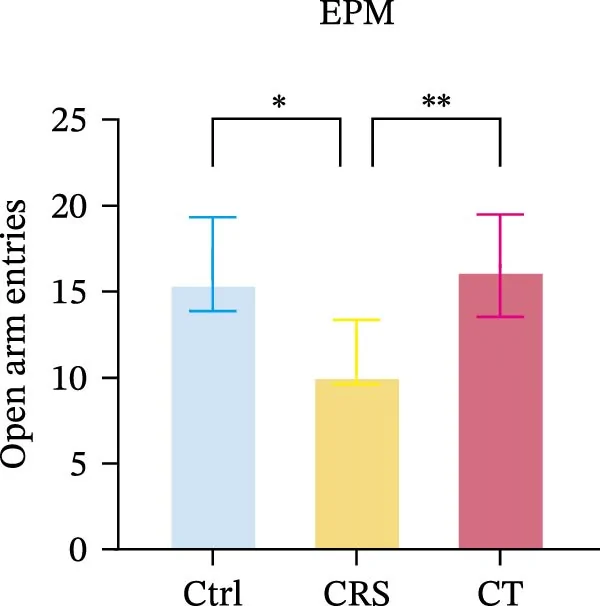
(F)

**Figure:**
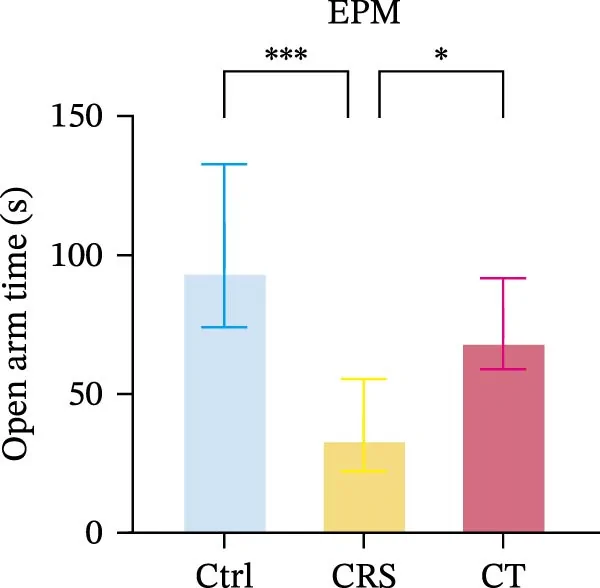
(G)

**Figure:**
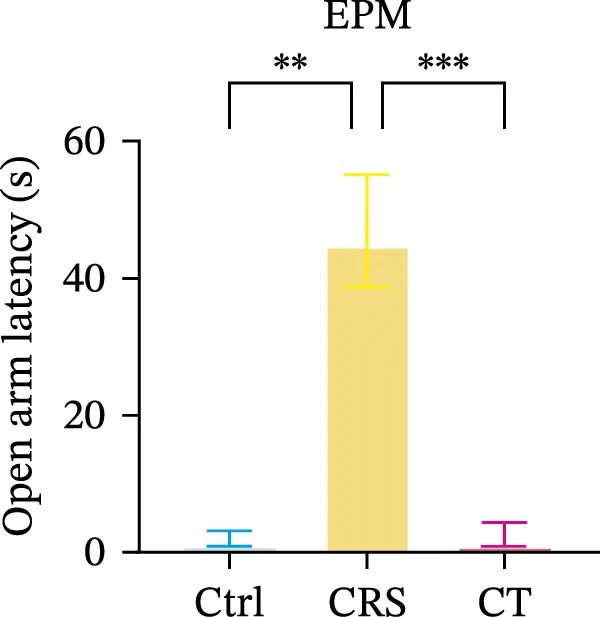
(H)

**Figure:**
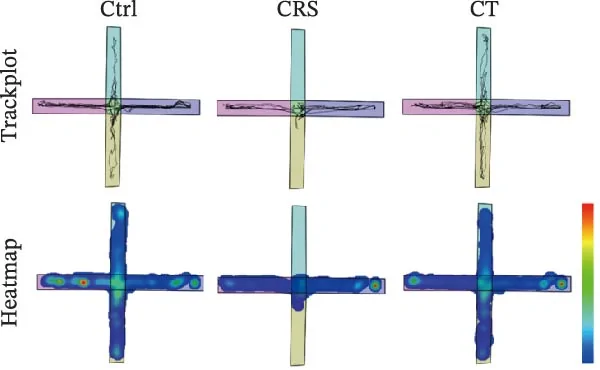
(I)

**Figure:**
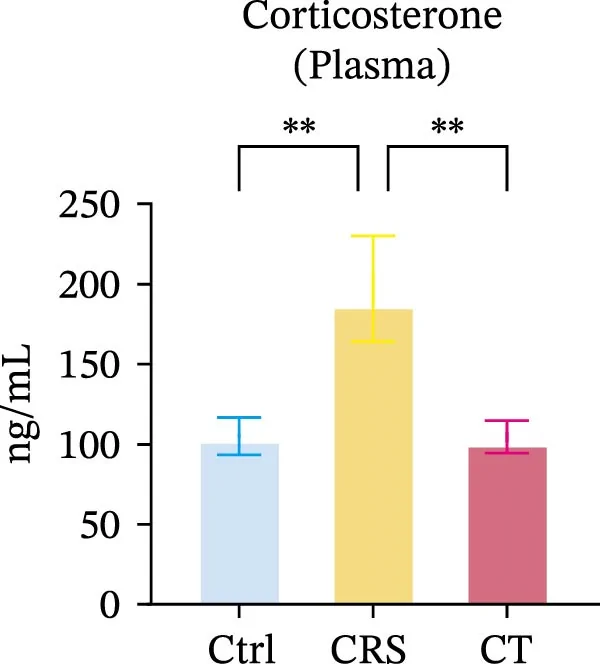
(J)

### 2.2. Treadmill Exercise Attenuates CRS‐Related Neuron Injury by Suppressing the Inflammatory Response

To further explore the neuroprotective effects of exercise, we evaluated neuronal integrity and inflammatory responses in the three experimental groups. Nissl staining revealed substantial neuronal damage in the hippocampus of CRS mice, characterized by reduced Nissl body density, disordered cell arrangement, and the presence of karyopyknotic nuclei (Figure 2A). Treadmill exercise significantly mitigated these alterations, restoring Nissl body density to near‐control levels. Concurrently, immunofluorescence analysis of Iba1‐positive cells revealed that CRS induced marked microglial activation (Figure 2B). This activation phenotype was significantly suppressed by treadmill exercise. Moreover, we analyzed dendritic spine density utilizing Golgi staining (Figure 2C). CRS significantly reduced total dendritic spine density in the brain compared to the Ctrl group. Treadmill exercise effectively ameliorated this reduction, restoring spine density to levels comparable to the Ctrl group (Figure 2D, one‐way ANOVA followed by Dunnett’s post hoc test, *p*  < 0.0001, *F* [2, 33] = 15.68). Morphological sub‐classification further revealed that CRS significantly decreased the density of both mature (Figure 2E, one‐way ANOVA followed by Dunnett’s post hoc test, *p* = 0.0004, *F* [2, 33] = 9.975) and immature (Figure 2F, one‐way ANOVA followed by Dunnett’s post hoc test, *p*  < 0.0001, *F* [2, 33] = 26.15) spines. Treadmill exercise intervention significantly increased the density of both spine subtypes in CRS‐exposed animals, counteracting the stress‐induced deficits across classifications (Figure 2C,E,F). We then used real‐time quantitative polymerase chain reaction (RT‐qPCR) to measure the expression of pro‐ and anti‐inflammatory cytokines in peripheral blood mononuclear cell (PBMC) and hippocampal tissues. Compared to the Ctrl group, mRNA levels of proinflammatory cytokines, such as IL‐6 and IL‐1β (Figure 2G, one‐way ANOVA followed by Dunnett’s post hoc test, *p* = 0.01, *F* [2, 8] = 8.652; Figure 2H, one‐way ANOVA followed by Dunnett’s post hoc test, *p*  < 0.0001, *F* [2, 9] = 39.36; Figure 2K, one‐way ANOVA followed by Dunnett’s post hoc test, *p* = 0.0007, *F* [2, 10] = 16.69; Figure 2L, one‐way ANOVA followed by Dunnett’s post hoc test, *p* = 0.0021, *F* [2, 9] = 13.19) were significantly elevated in the CRS group. Conversely, mRNA levels of anti‐inflammatory cytokines, including TGF‐β and IL‐10, were significantly reduced in the CRS group (Figure 2I, one‐way ANOVA followed by Dunnett’s post hoc test, *p* = 0.0002, *F* [2, 8] = 28.45; Figure 2J, one‐way ANOVA followed by Dunnett’s post hoc test, *p*  < 0.0001, *F* [2, 9] = 33.14; Figure 2M, Kruskal–Wallis test followed by Dunn’s post hoc test, *p* = 0.0012, Kruskal–Wallis statistical magnitude = 8.769; Figure 2N, one‐way ANOVA followed by Dunnett’s post hoc test, *p* = 0.0339, *F* [2, 7] = 5.703). In the CT group, mRNA levels of IL‐6 and IL‐1β were significantly lower than in the CRS group (Figure 2G,H,K,L). Treadmill exercise also significantly increased TGF‐β and IL‐10 expression in PBMC of the CT group. A similar trend was observed in hippocampal tissue, but it did not reach statistical significance (Figure 2I,J,M,N). In addition, western blot analysis of hippocampal tissue showed that phosphorylation of ERK1/2 was increased in CRS mice, an effect that was reversed by treadmill exercise (Figure 2O). These results demonstrate that treadmill exercise ameliorates neuronal injury and suppresses excessive neuroinflammation in the CRS model.

Figure 2Treadmill exercise mitigates CRS‐induced neuronal damage and excessive neuroinflammation. (A) Representative images of Nissl staining of the hippocampus; (B) representative images of lba1^+^ microglia immunofluorescent staining; (C) representative images of Golgi staining of dendritic spines; (D) density of total dendritic spines in Golgi staining; (E) density of mature dendritic spines in Golgi staining; (F) density of immature dendritic spines in Golgi staining (*n* = 4/group,  ^∗^ *p*  < 0.05,  ^∗∗^ *p*  < 0.01,  ^∗∗∗^ *p*  < 0.001,  ^∗∗∗∗^ *p*  < 0.0001); (G–J) after behavioral tests, the PBMC were collected for RT‐qPCR analysis. (G) IL‐1β; (H) IL‐6; (I) IL‐10; (J) TGF‐ β. (K–N) After behavioral tests, the hippocampus tissue was collected for RT‐qPCR analysis. (K) IL‐1β; (L) IL‐6; (M) IL‐10; (N) TGF‐β; (O) representative western blotting image of the hippocampus. Each experiment was independently repeated three times. *n* = 3–5/group,  ^∗^ *p*  < 0.05,  ^∗∗^ *p*  < 0.01,  ^∗∗∗^ *p*  < 0.001,  ^∗∗∗∗^ *p*  < 0.0001, and ns, no significant difference. One‐way ANOVA (D, E, F, G, H, I, J, K, L, N) followed by Dunnett’s post hoc test. Kruskal–Wallis test (M) followed by Dunn’s post hoc test.

**Figure:**
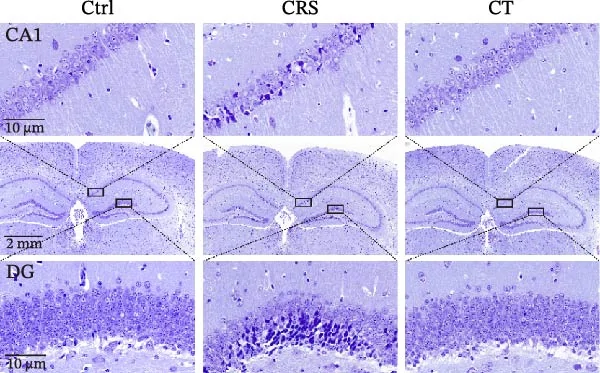
(A)

**Figure:**
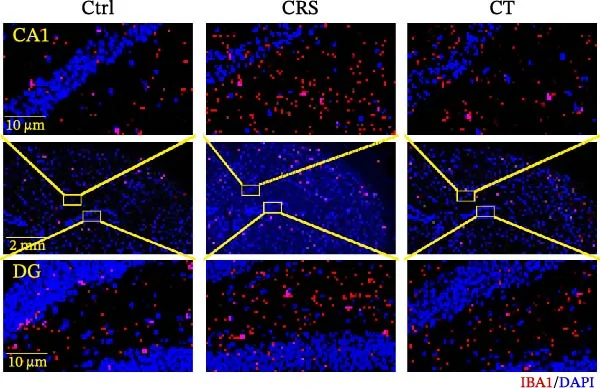
(B)

**Figure:**
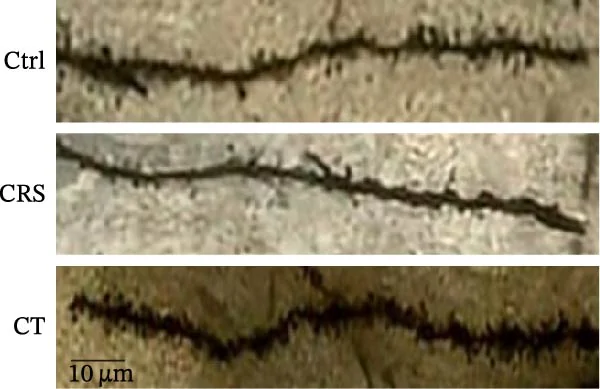
(C)

**Figure:**
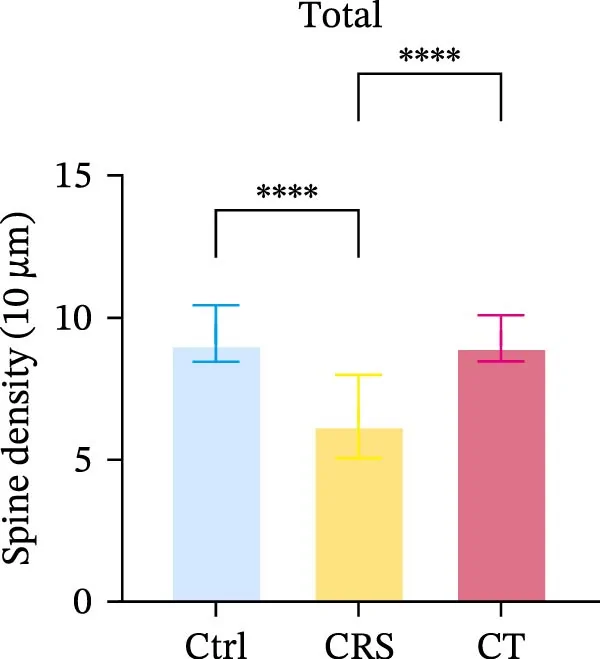
(D)

**Figure:**
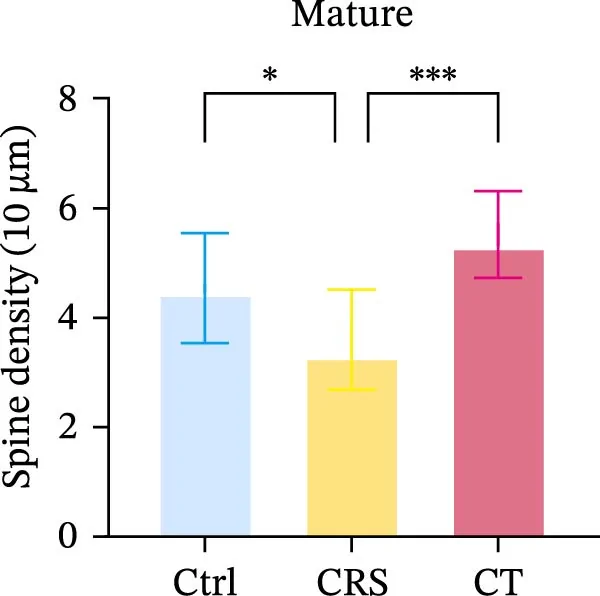
(E)

**Figure:**
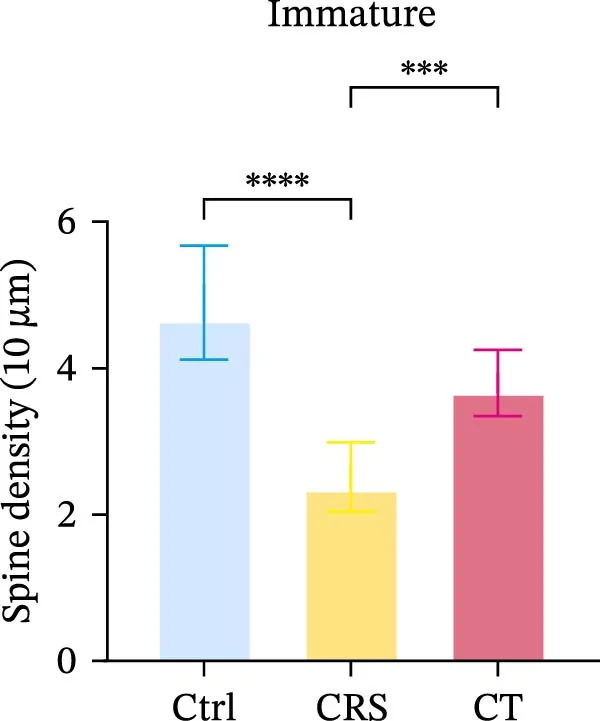
(F)

**Figure:**
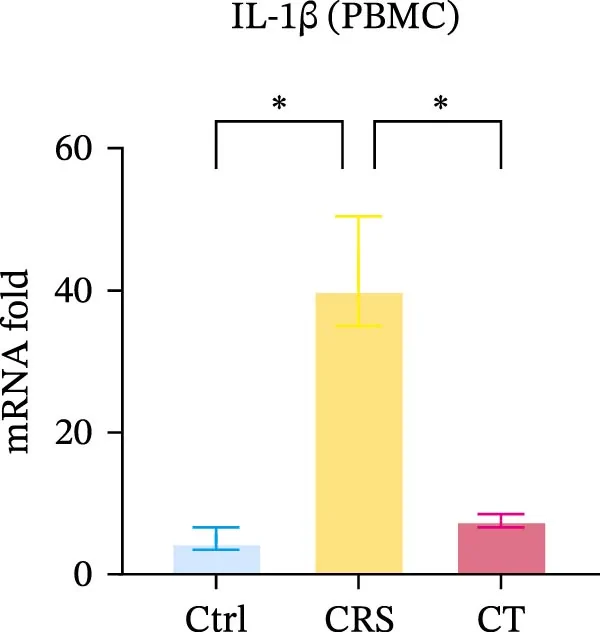
(G)

**Figure:**
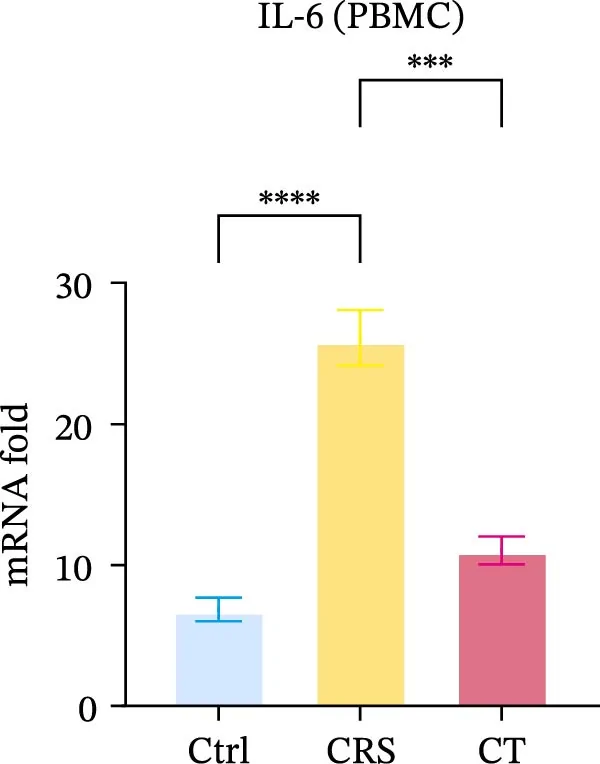
(H)

**Figure:**
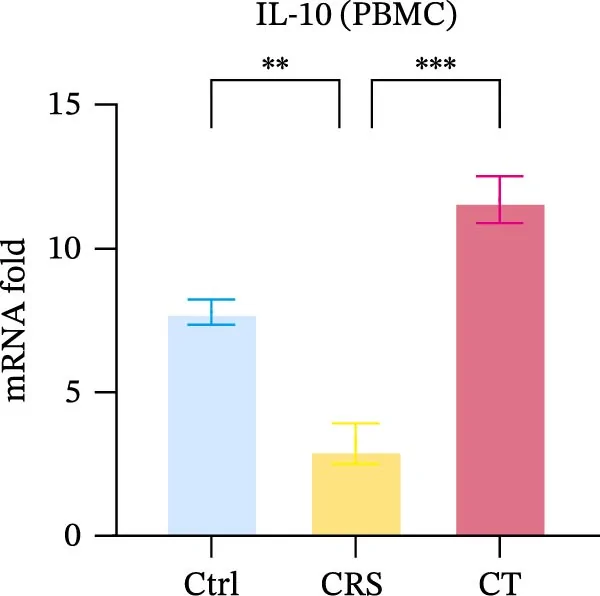
(I)

**Figure:**
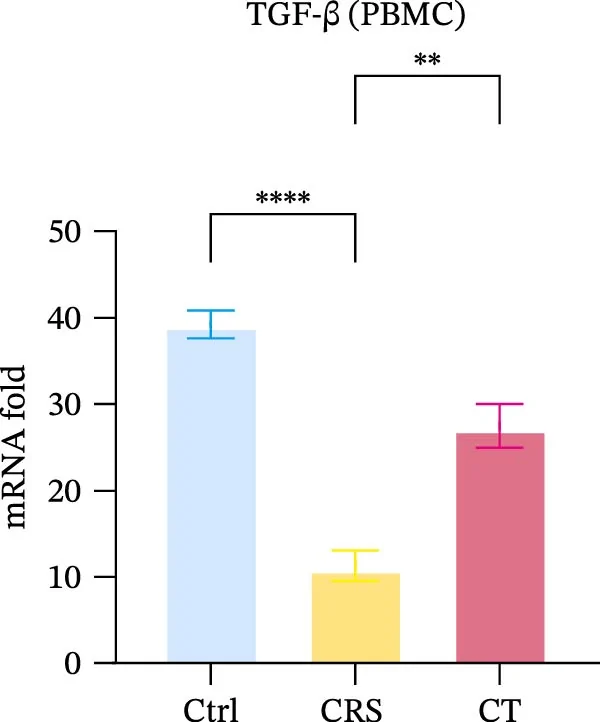
(J)

**Figure:**
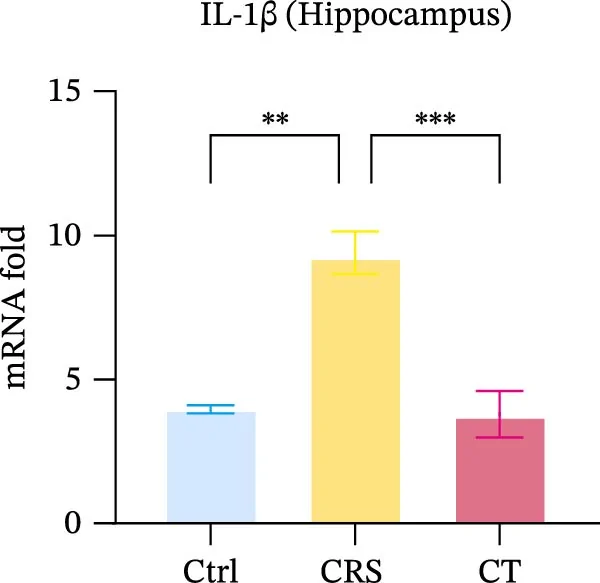
(K)

**Figure:**
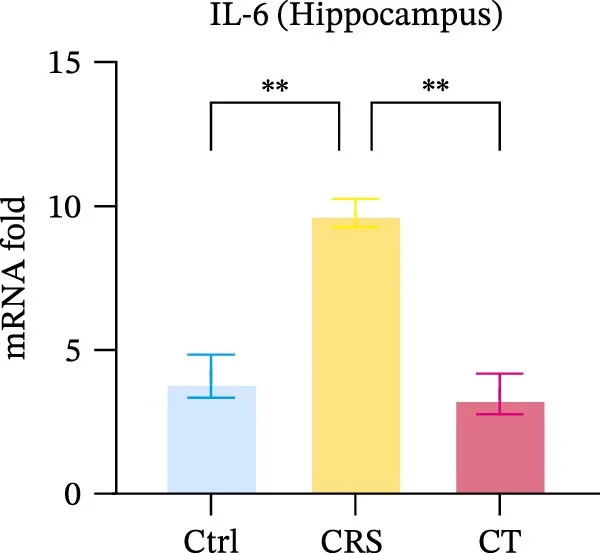
(L)

**Figure:**
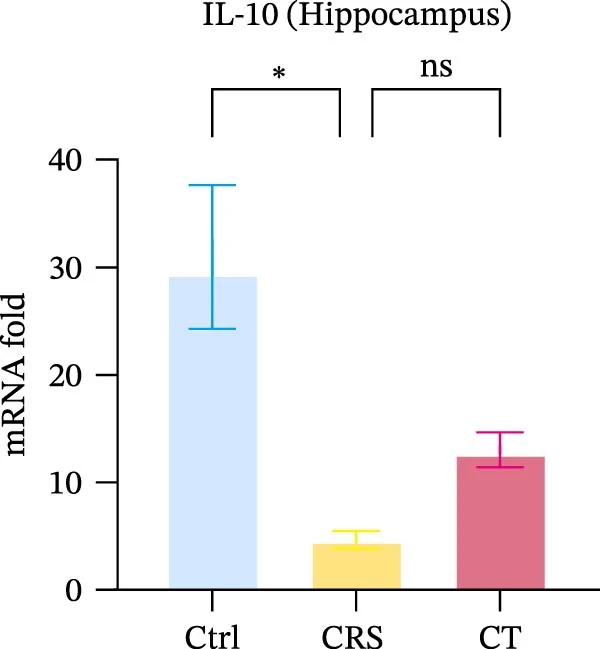
(M)

**Figure:**
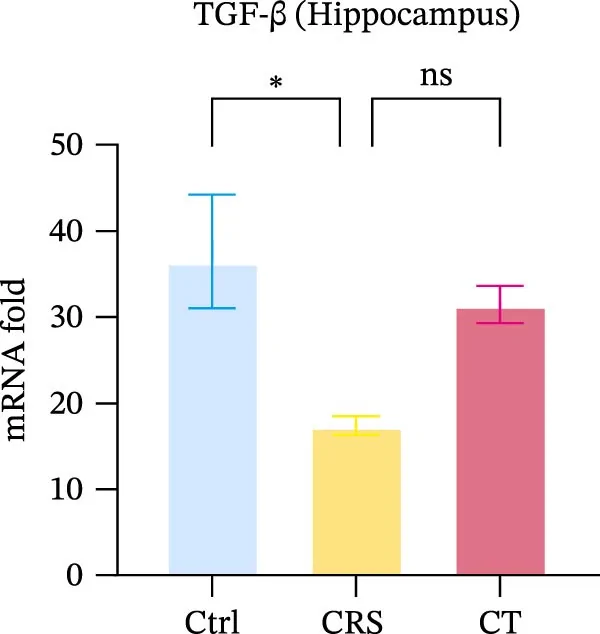
(N)

**Figure:**
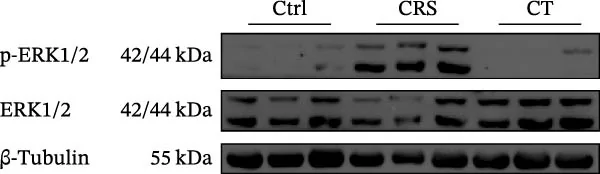
(O)

### 2.3. Treadmill Exercise Suppresses CRS‐Induced GDF15 Expression

Our previous results suggested that exercise alleviates depressive‐like behaviors and reduces neuroinflammation in CRS mice. To elucidate the underlying molecular mechanisms, we conducted transcriptome sequencing analysis on PBMC from the Ctrl, CRS, and CT groups. Differentially expressed genes (DEGs) were identified using thresholds of |fold change| of ≥2 and an adjusted *p*‐value (padj) of ≤0.05. Compared to the CRS group, the CT group exhibited 1108 DEGs (996 upregulated, 112 downregulated). Conversely, the CRS group showed 1375 DEGs (376 upregulated, 999 downregulated) compared to the Ctrl group (Figure 3A). Volcano plots visualizing these DEG distributions are shown in Figure 3B.

Figure 3GDF15 as a pivotal mediator of stress‐induced nerve injury in CRS mice. (A) Differential gene expression (DEG) counts across groups (fold change ≥ 2, *p*‐value ≤ 0.05, and padj ≤ 0.05). (B) volcano plots of DEGs among three groups of mice; (C) representative images of GDF15 immunofluorescent staining; (D) the expression of GDF15 in mouse hippocampus was detected by western blot; (E) the grayscale analysis results of the western blotting image ( ^∗^ *p*  < 0.05,  ^∗∗^ *p*  < 0.01); (F) plasma GDF15 levels (pg/mL) were analyzed by enzyme‐linked immunosorbent assay (ELISA) after the behavior tests; (G) GO enrichment bar plots highlighting immune‐related pathways. Each experiment was independently repeated three times. *n* = 6/group,  ^∗^ *p*  < 0.05,  ^∗∗^ *p*  < 0.01. One‐way ANOVA (F) followed by Dunnett’s post hoc test. Welch’s ANOVA (E) followed by Dunnett’s T3 post hoc test.

**Figure:**
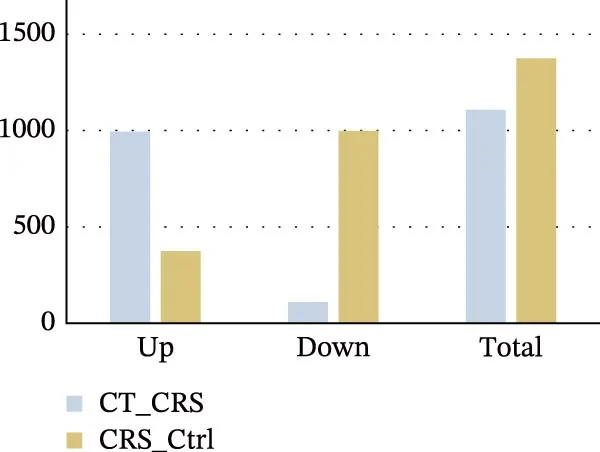
(A)

**Figure:**
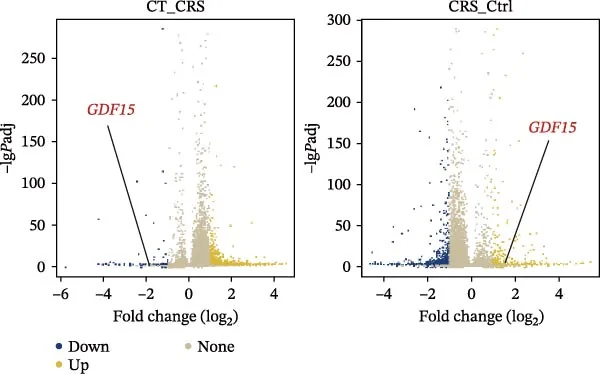
(B)

**Figure:**
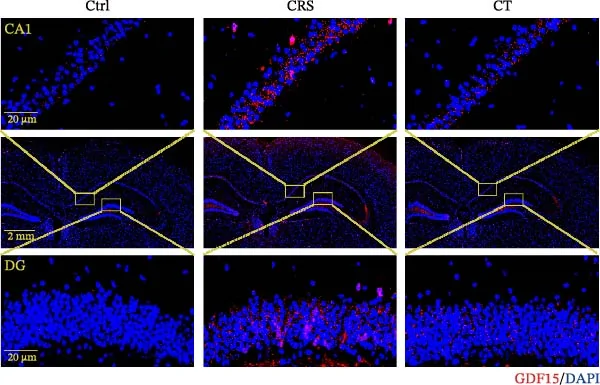
(C)

**Figure:**
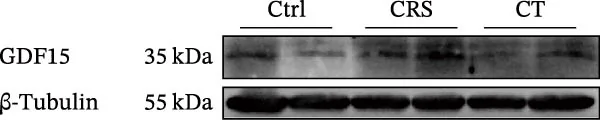
(D)

**Figure:**
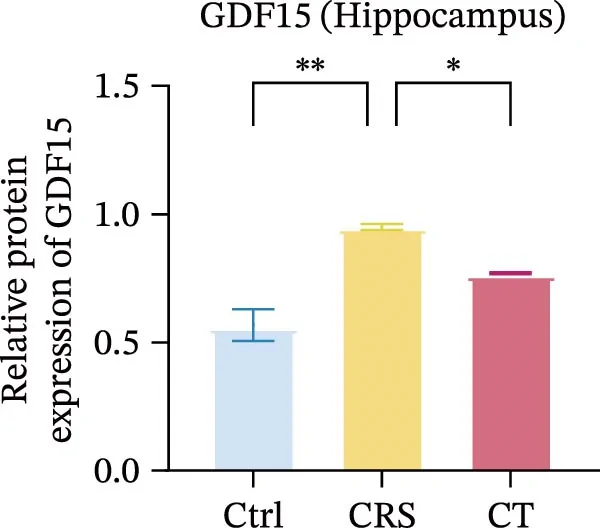
(E)

**Figure:**
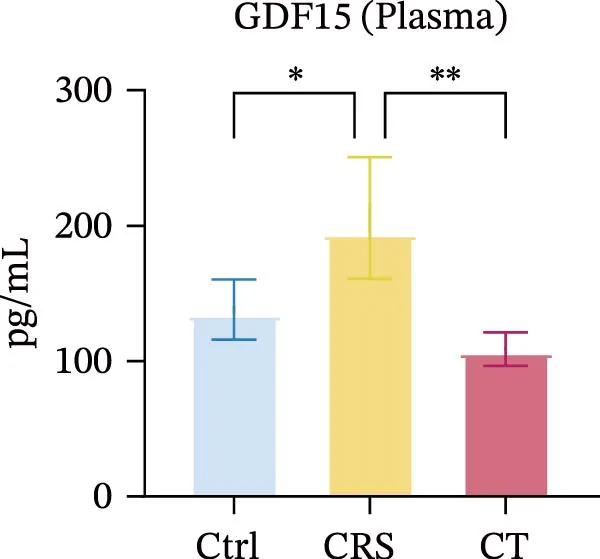
(F)

**Figure:**
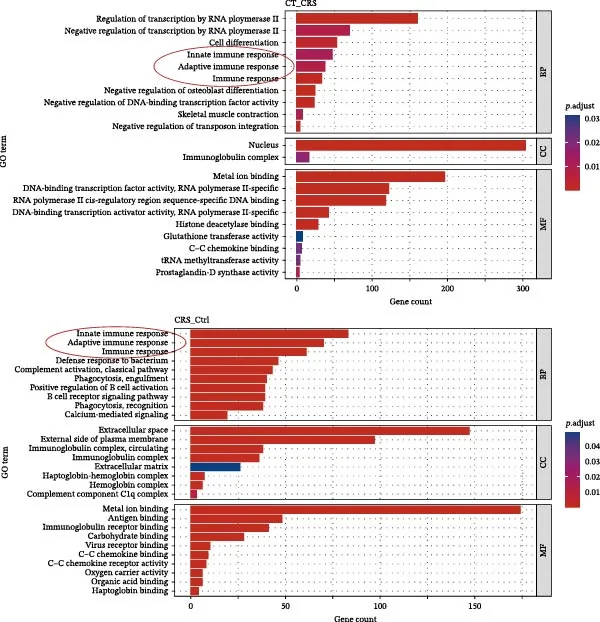
(G)

Notably, among these DEGs, GDF15 expression was upregulated in the CRS versus Ctrl comparison but downregulated in the CT versus CRS comparison. This pattern indicates that CRS elevates GDF15 expression, while exercise intervention mitigates this increase. We next validated this finding in mouse tissues. Immunofluorescence staining of mouse brain tissue confirmed elevated GDF15 expression in the CRS group, which was attenuated by treadmill exercise (CT group) (Figure 3C). Western blot analysis of hippocampal tissue lysates corroborated these findings. CRS significantly increased GDF15 protein levels compared to the Ctrl group, and treadmill exercise effectively normalized this elevation (Figure 3D,E, Welch’s ANOVA followed by Dunnett’s T3 post hoc test, *p* = 0.0244, *W* [2.000,1.768] = 58.11). Enzyme‐linked immunosorbent assay (ELISA) of peripheral plasma assays corroborated that exercise reduced CRS‐induced circulating GDF15 levels (Figure 3F, one‐way ANOVA followed by Dunnett’s post hoc test, *p* = 0.0027, *F* [2, 15] = 8.977), consistent with the PBMC transcriptome data. Together, these consistent results demonstrate that treadmill exercise mitigates the stress‐induced increase of GDF15 both peripherally and in the hippocampus. To probe the functional role of GDF15, we employed GDF15 whole‐body knockout (GDF15^-^/^-^) mice subjected to CRS of 14 days, followed by behavioral tests and assessment of hippocampal neuroinflammation and ERK 1/2 phosphorylation. Notably, in stark contrast to wild‐type mice, GDF15^-^/^-^ mice exhibited resilience to stress‐induced behavioral deficits and reduced neuroinflammation (Figure S1).

We next performed Gene Ontology (GO) functional enrichment analysis on the DEGs using the ClusterProfiler software. This analysis aimed to identify biological functions and pathways significantly associated with the DEGs, thereby elucidating potential molecular mechanisms. The GO enrichment analysis revealed the primary biological functions associated with the candidate genes. The top 30 most significantly enriched GO terms (or all terms if fewer than 30 were identified) were selected and visualized in bar charts (Figure 3G). The analysis revealed that numerous DEGs were enriched in immune response pathways. This suggests that specific immune‐related DEGs may mediate the ameliorative effects of exercise on depressive‐like behaviors in CRS mice. The observed changes in pro‐ and anti‐inflammatory cytokines following CRS and treadmill exercise (Figure 2) support the hypothesis that exercise promotes immune homeostasis. However, the role of GDF15 in the regulation of neuroinflammation remains to be determined.

### 2.4. GDF15 Promotes Neuroinflammation by Activating the ERK1/2 Pathway

To investigate the role of GDF15 in neuroinflammation, an in vitro depression model was established by stimulating microglia cells (BV‐2) with LPS [31, 32]. First, treating BV‐2 cells with increasing doses of LPS for 6 h resulted in an upregulation *GDF15* expression (Figure 4A, one‐way ANOVA followed by Dunnett’s post hoc test, *p*  < 0.0001, *F* [3,8] = 113.3). BV‐2 cells produce high levels of *GDF15* in response to hyperinflammatory stimuli, which may partly account for the high *GDF15* expression in CRS mice.

Figure 4GDF15 exacerbates LPS‐induced proinflammatory responses in BV‐2 cells. (A) BV‐2 cells were treated with LPS (0, 500, 1000, and 2000 ng/mL) for 6 h, then the GDF15 mRNA fold change was detected by RT‐qPCR; (B) BV‐2 cells were treated with rmGDF15 (1, 10, and 100 ng/mL) for 24 h, then the expression of p‐ERK 1/2 and ERK 1/2 was detected by western blot; (C) BV‐2 cells were transfected with shRNA‐GDF15, then the expression of p‐ERK 1/2, ERK 1/2 and GDF15 was detected by western blot; (D) BV‐2 cells were treated with LPS (500 ng/mL) in the presence of rmGDF15 (100 ng/mL) for 6 h, then the expressions of p‐ERK 1/2 and ERK 1/2 were detected by western blot; (E) BV‐2 cells were treated with LPS (500 ng/mL) in the presence of transfection of shRNA‐GDF15, then the expressions of p‐ERK 1/2, ERK 1/2, and GDF15 were detected by western blot; (F–H) BV‐2 cells were treated with rmGDF15 (100 ng/mL), selumetinib (10 μm) and LPS (500 ng/mL) for 6 h, then the inflammatory factors mRNA fold change was detected by RT‐qPCR. (F) IL‐1β; (G) IL‐6; (H) TNF‐α. Each experiment was independently repeated three times. *n* = 3/group,  ^∗^ *p*  < 0.05,  ^∗∗^ *p*  < 0.01, ^∗∗∗^ *p*  < 0.001,  ^∗∗∗∗^ *p*  < 0.0001, and ns, no significant difference. One‐way ANOVA (A, F, G, H) followed by Dunnett’s post hoc test.

**Figure:**
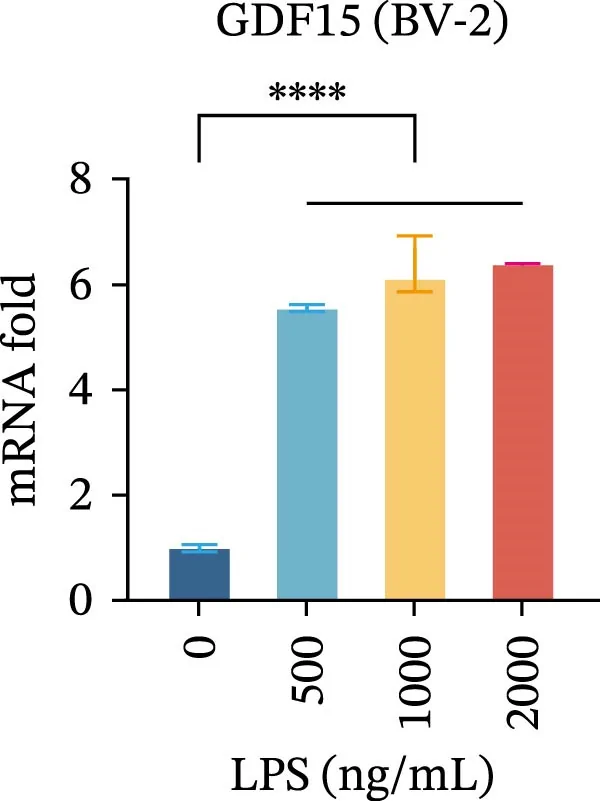
(A)

**Figure:**
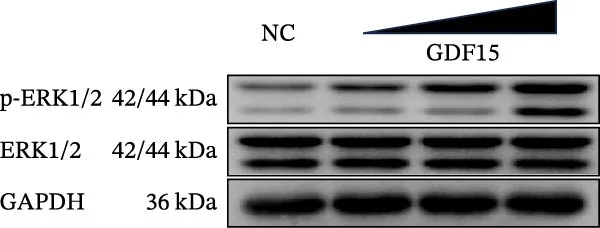
(B)

**Figure:**
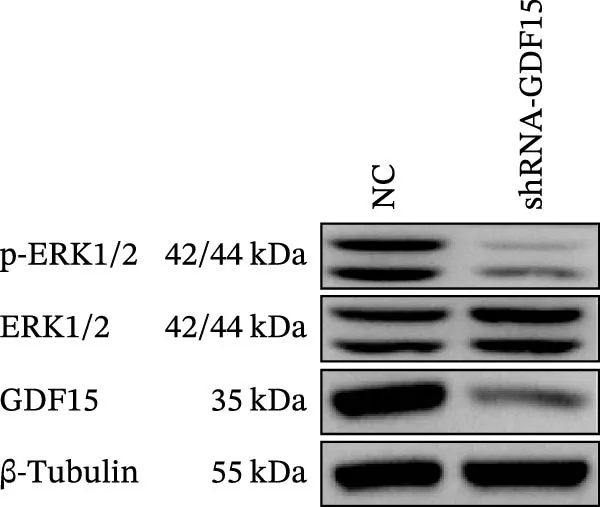
(C)

**Figure:**
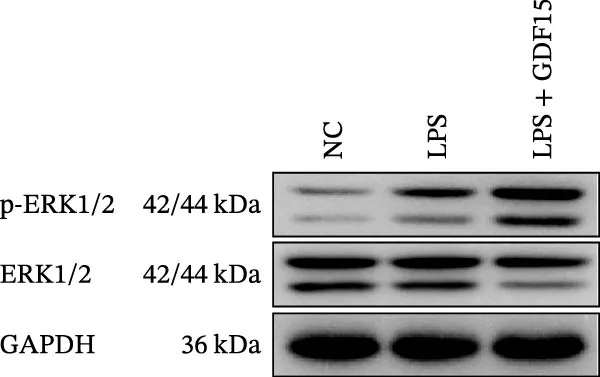
(D)

**Figure:**
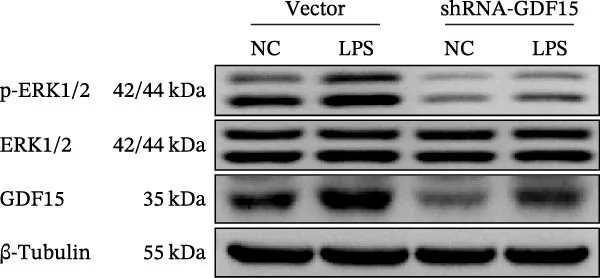
(E)

**Figure:**
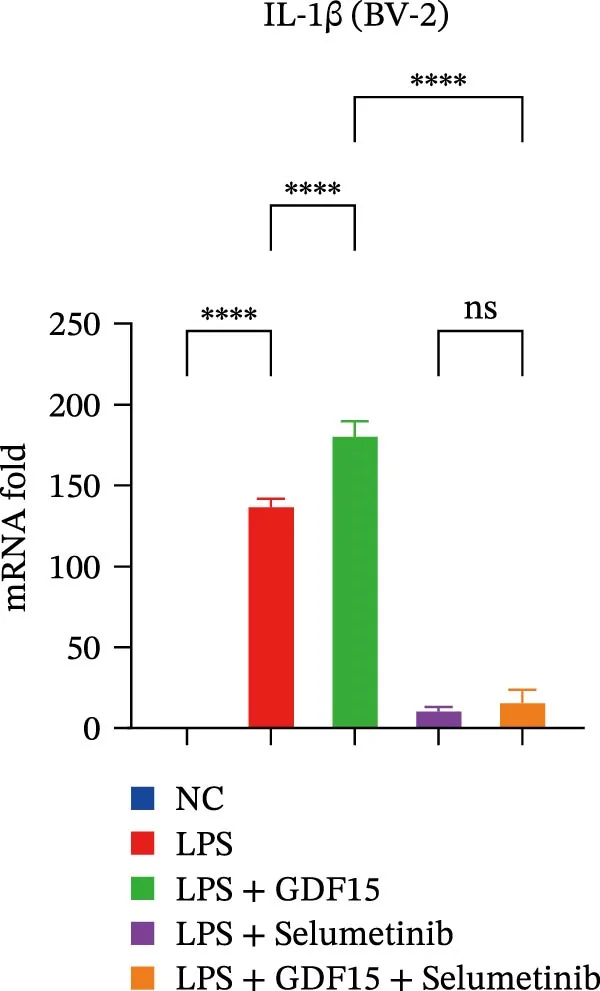
(F)

**Figure:**
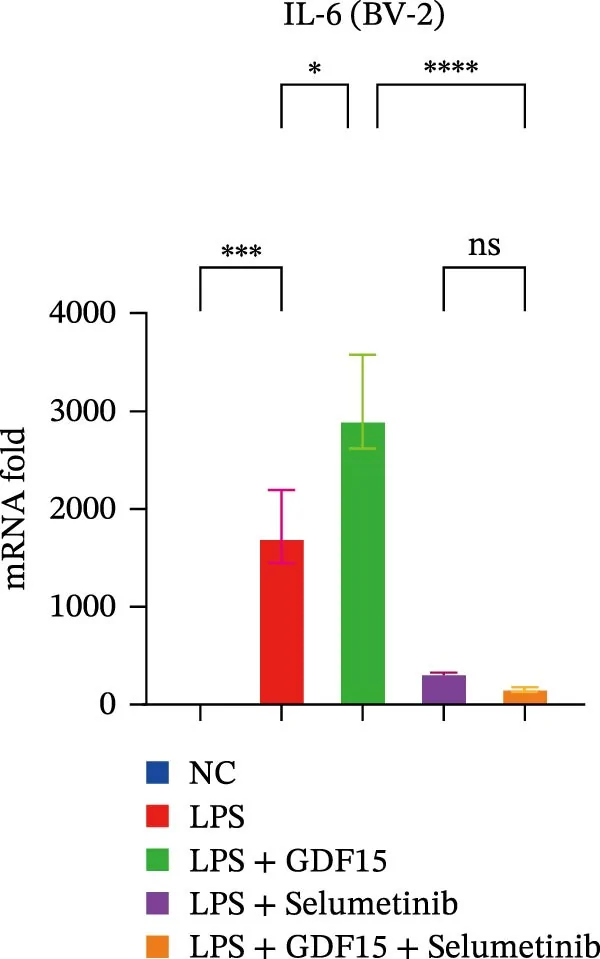
(G)

**Figure:**
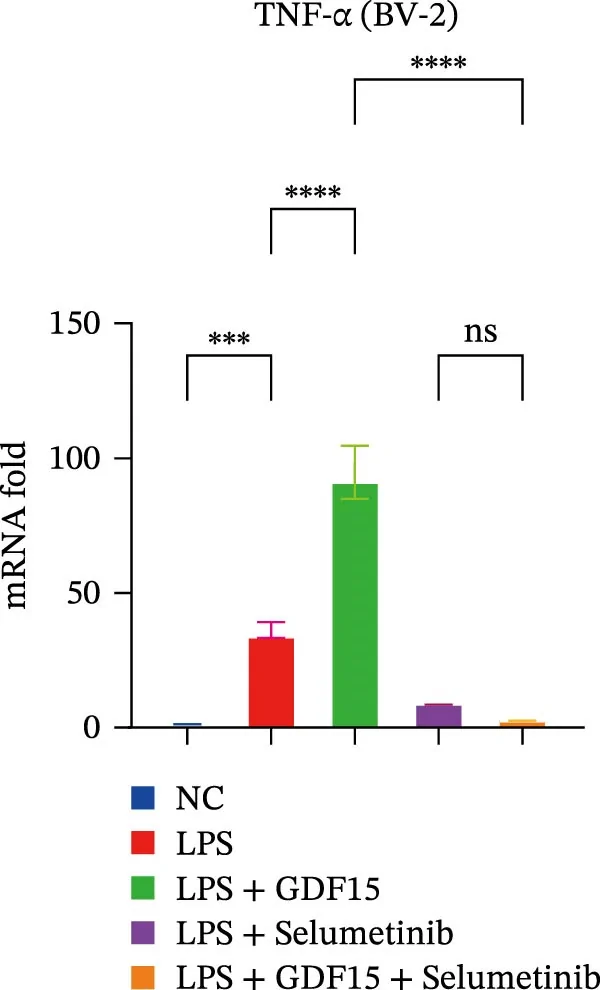
(H)

Recombinant mouse GDF15 (rmGDF15) protein potentiated the ERK signaling pathway in a dose‐dependent effect in BV‐2 cells (Figure 4B). Conversely, knockdown of GDF15 using shRNA reduced the phosphorylation level of ERK 1/2 in BV‐2 cells (Figure 4C). In addition, rmGDF15 can further enhance LPS‐induced ERK 1/2 phosphorylation (Figure 4D). GDF15 knockdown led to reduced responsiveness of microglia to LPS stimulation, suggesting a synergistic effect between GDF15 and LPS in inducing ERK activation (Figure 4E). These in vitro findings are consistent with our in vivo observations, confirming the involvement of GDF15 in regulating ERK signaling. To further investigate the relationship between GDF15, ERK, and inflammation, BV‐2 cells were pretreated with rmGDF15 protein (100 ng/mL) and the ERK pathway inhibitor selumetinib (10 μm) for 30 min, followed by LPS (500 ng/mL) stimulation for 6 h. RT‐qPCR analysis of proinflammatory cytokines demonstrated that rmGDF15 pretreatment synergistically increased LPS‐induced expression of IL‐1β, IL‐6, and TNF‐α, an effect that was abolished by selumetinib cotreatment (Figure 4F, one‐way ANOVA followed by Dunnett’s post hoc test, *p*  < 0.0001, *F* [4, 9] = 756.4; Figure 4G, one‐way ANOVA followed by Dunnett’s post hoc test, *p*  < 0.0001, *F* [4, 10] = 34.19; Figure 4H, one‐way ANOVA followed by Dunnett’s post hoc test, *p*  < 0.0001, *F* [4, 10] = 99.27). These results indicate that GDF15 exacerbates the LPS‐induced inflammatory response in BV‐2 cells by promoting the phosphorylation of ERK1/2. These in vitro findings support a causal role for GDF15 in driving neuroinflammation. The ERK pathway plays a critical role in GDF15‐mediated neuroinflammation. Collectively, these data suggest that exercise may attenuate depression‐like behaviors by suppressing GDF15/ERK‐induced neuroinflammation.

## 3. Discussion

In summary, our findings demonstrate that treadmill exercise effectively ameliorates depressive‐like behaviors in CRS mice, alleviating neuronal damage and excessive inflammatory responses in both the periphery and the central neuroimmune systems. To elucidate the underlying mechanisms, we performed transcriptomic analysis. This analysis revealed a critical role for GDF15, a stress‐responsive factor, in mediating the antidepressant effects of exercise. Subsequent in vivo data indicated that exercise normalizes the CRS‐induced upregulation of GDF15 in both central and/or peripheral neuroimmune systems. Complementary in vitro studies showed that GDF15 exacerbates LPS‐induced inflammation in microglia by promoting ERK activation. Collectively, these findings suggest that treadmill exercise can reduce the expression of GDF15, subsequently decrease the phosphorylation level of ERK, improve neuroinflammation, and exert an antidepressant effect.

Exercise is gaining recognition as a nonpharmacological therapy for depression, with a growing body of studies elucidating its therapeutic effects and underlying mechanisms. For example, running exercise has been associated with reduced depressive symptoms and enhanced white matter integrity [33, 34]. Our result that treadmill exercise reversed CRS‐induced behavioral deficits further supports the beneficial effects of exercise on depression. Numerous randomized controlled trials and systematic reviews have explored the impact of exercise on depression. As early as 1984, McCann and colleagues investigated the effect of aerobic exercise on depression. They randomly assigned 43 patients with depression to aerobic exercise, relaxation therapy, or no‐treatment group, providing some of the first controlled evidence for the antidepressant effects of exercise [35]. Clinical practice guidelines in the United States, United Kingdom, and Australia recommend physical activity as part of treatment for depression [36]. Exercise represents a viable nonpharmaceutical treatment with benefits that may persist beyond the cessation of treatment—an advantage over some pharmacotherapies. While the efficacy of exercise is well‐established, its benefits have been predominantly attributed to mechanisms such as enhanced neurogenesis [37], upregulation of brain‐derived neurotrophic factor (BDNF) [38], or normalization of the HPA axis [39]. Our work identifies a novel neuroimmune axis centered on GDF15 suppression as a fundamental mechanism. Crucially, our transcriptomics analysis revealed that exercise reverses CRS‐induced upregulation of GDF15, a cytokine previously unrecognized in depression pathophysiology. GO enrichment and our in vitro data highlight its pivotal role in immune processes. This represents a divergence from the classical neurotrophic hypothesis by positioning the resolution of neuroinflammation as a driver of exercise’s benefits, a paradigm shift supported by growing evidence linking peripheral inflammation to depression [40]. Our data thus establish GDF15 as a critical regulator that converts exercise into neuroimmune homeostasis.

It is known that proinflammatory responses contribute to the pathogenesis of depression. We examined the expression of key proinflammatory cytokines, including IL‐1β and IL‐6, and anti‐inflammatory cytokines, TGF‐β and IL‐10, in both the brain (hippocampus) and the peripheral immune system (PBMCs). In our model, treadmill exercise decreased the expression of proinflammatory cytokines and increased anti‐inflammatory cytokines. Immunofluorescence analysis also demonstrated that treadmill exercise inhibited the activation of microglia in the brain. Current consensus is that exercise broadly suppresses neuroinflammation, for example, by dampening NF‐κB signaling [41] or enhancing anti‐inflammatory cytokines [42]. However, our study identifies GDF15 as a selective upstream activator of proinflammatory microglial responses via ERK phosphorylation. In vitro, GDF15 acted synergistically with LPS to cause neuroinflammation, an effect that was abolished by selumetinib, confirming phospho‐ERK as the critical effector. Significantly, exercise reduced GDF15 levels and normalized p‐ERK/ERK ratios in CRS mice, directly linking exercise to suppression of this pathway. Our findings move beyond generic models of inflammation suppression by revealing a ligand‐specific circuit (GDF15‐ERK‐cytokines). This mechanism helps explain the precision of exercise’s anti‐inflammatory action within the neuroimmune axis.

There is increasing evidence that GDF15 is involved in various diseases, often through its proinflammatory effects. For example, GDF15 upregulates IL‐6 to promote prostate carcinoma tumorigenesis, while its deficiency reduces vascular injury‐induced IL‐6 upregulation [43]. In response to mechanical stress in periodontal ligament fibroblasts (PdLF), GDF15 acts as an important autocrine proinflammatory regulator that exerts its effects in a manner [44]. Furthermore, GDF15 expressed for long periods of time in chronic pathologic processes (e.g., obesity and aging) can drive a chronic low‐grade inflammatory state [45]. Changes in neuroplasticity are often accompanied by alterations in cellular signaling, in which the MAPK pathway plays a crucial role in the modification of neural plasticity. This pathway is involved in neuroinflammation, neuronal apoptosis, and the pathophysiology of depression [21, 46]. ERK1/2 is a member of the MAPK family, which is involved in cell division and growth, signaling transmission, proliferation, and other physiological functions [47]. In this study, we observed that GDF15 expression was upregulated following both CRS and LPS stimulation. Subsequently, GDF15 activates the MAPK pathway by promoting ERK phosphorylation. As a result, CRS‐induced GDF15 contributes to an excessive inflammatory response, as GDF15 treatment enhances the expression of IL‐1β, IL‐6, and TNF‐α in microglia. Conversely, exercise‐mediated reduction of GDF15 levels was closely associated with the amelioration of depressive behaviors. This indicates that GDF15 can integrate stress, immunity, and neuronal damage and serve as a modifiable target. Our work therefore moves beyond establishing GDF15 as a mere biomarker [48] by delineating its mechanism of action, thereby integrating the axes of stress response, immune dysregulation, and neuronal damage in depression.

**Figure 5:**
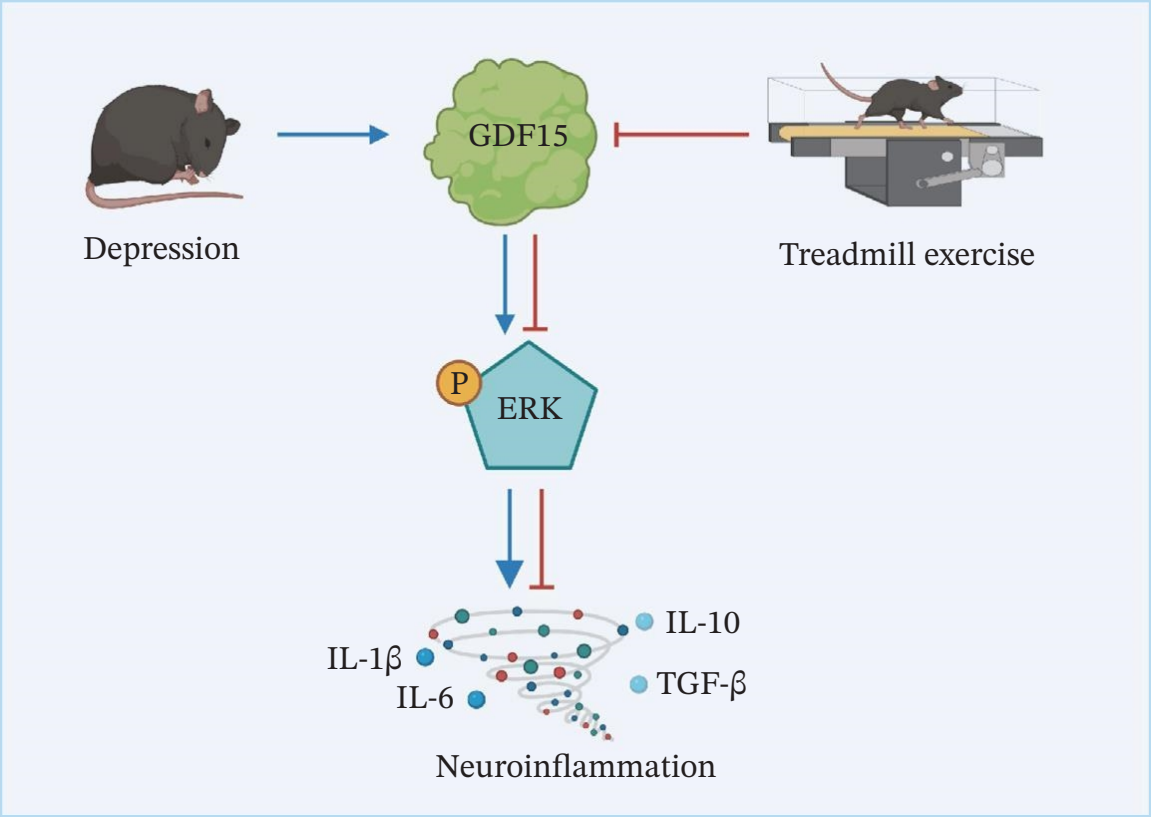
Working model of the mechanism by which treadmill exercise regulates depression. In this model, CRS enhances the activation of the GDF15‐ERK pathway induced neuroinflammation, which in turn promotes depression. On the contrary, treadmill exercise can suppress the overexpression of GDF15, thus improving neural inflammation and exerting an antidepressant effect (created in BioRender, https://BioRender.com/rg8riuc).

In conclusion, our study identifies a novel mechanism through which exercise ameliorates depression. Specifically, treadmill exercise reduces CRS‐induced GDF15 expression, thereby inhibiting the GDF15/ERK pathway, attenuating neuroinflammation, and ultimately alleviating depression symptoms. These findings position GDF15 as a promising therapeutic target for depression. A schematic diagram of how exercise suppresses the GDF15‐ERK inflammatory pathway was shown in Figure 5.

## 4. Material and Methods

### 4.1. Animal Experiment

Six‐week‐old male C57BL/6J Nifdc mice weighing 19 ± 1 g were obtained from SPF (Beijing) Biotechnology Co., Ltd and maintained in an SPF facility with constant temperature (25 ± 1°C), constant humidity (55% ± 5%), and a 12 h light–dark cycle (light period: 7:00–19:00). During the experiment, each cage housed no more than five mice with ad libitum access to food and water. Bedding was replaced twice weekly. All procedures complied with protocols approved by the Animal Experiment Ethics Committee of the Institute of Basic Medical Sciences, Beijing (License Number IACUC‐DWZX‐2022‐546). The sample size was determined by the 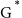 Power software using preliminary experimental results.

After 1 week of adaptation, the mice were randomly divided into three groups (*n* = 8 per group): (1) Ctrl group: without any treatment. (2) CRS group: we constructed restraint devices for mice using 50 mL conical tubes (Servicebio, #EP‐5000‐BJ). Mice were subjected to restraint stress using these devices for 6 h daily (10:00–16:00) for 14 consecutive days. (3) CRS and Treadmill exercise group (CT group): Mice underwent treadmill (SansBio, # SA101C) exercise from 8:00 to 9:00 a.m. at a speed of 12 m/min, followed by a 1‐h rest period. Subsequently, they were subjected to 6 h of restraint stress concurrently with the CRS group for 14 consecutive days (Figure 1A).

### 4.2. Behavioral Tests

Mice were treated with CRS and treadmill exercise for 14 days. The behavioral tests were conducted from day 15. On the 15th day, all mice participated in the EPM test, followed by the TST on the 16th day, and the FST on the 17th day. All animals underwent the three behavioral tests.

#### 4.2.1. EPM

The EPM apparatus consisted of two open arms (50 cm × 10 cm) and two closed arms (50 cm × 10 cm), elevated ~50 cm above the floor, intersecting at a central platform (10 cm × 10 cm). The closed arms were surrounded by 30 cm high walls. Mice were relocated to the testing room at least 2 h before testing for acclimatization. At the beginning of each test session, a mouse was gently placed on the central platform facing an open arm. Behavior was recorded for 5 min, and the following parameters were analyzed: mean speed, latency to first enter an open arm, time spent in the open arms, and number of open arm entries, total entries (the sum of entries in closed and open arms), OE% (percentage of entries in open arms out of the total entries), and OT% (percentage of time in open arms out of the sum of time in closed and open arms). Following each test, the mouse was removed from the apparatus. The maze was thoroughly cleaned with 75% ethanol and allowed to dry completely before testing the next mouse.

#### 4.2.2. FST

The FST apparatus consisted of a transparent acrylic cylinder measuring 20 cm in diameter and 40 cm in height. Before the test, the cylinder was filled with water maintained at 22 ± 1°C to a depth of 20 cm. At the start of the test session, a mouse was placed into the cylinder. The test duration was 6 min. Behavior was recorded, and immobility time during the last 4 min was analyzed. Following each test, the mouse was removed from the cylinder. Any feces or other debris in the water were promptly removed using a fine mesh strainer net to prevent interference. If significant contamination occurred (e.g., due to excessive feces or urine), the water was completely replaced with fresh water maintained at 22 ± 1°C.

#### 4.2.3. TST

During the test, mice were secured by medical adhesive tape applied to the posterior third of the tail and suspended in an enclosed chamber. The animals were suspended such that their heads were positioned ~15 cm above the floor of the chamber. The test session lasted 6 min, and behavior was recorded from the front. Immobility time during the last 4 min of the test was analyzed. Following each test, the mouse was removed from the chamber. Similar to the EPM procedure, subsequent experiments were performed after thorough cleaning of the device.

### 4.3. Mice Euthanasia and Tissue Collection

Following behavioral testing, mice were euthanized by CO_2_ asphyxiation on the 18th day. Whole blood was drawn via cardiac puncture using heparin‐coated Vacutainer tubes. Within 10 min of collection, samples were centrifuged (2000 × g, 15 min, 4°C). Plasma aliquots (200 μL/tube) were flash‐frozen in liquid nitrogen and stored at −80°C for cytokine ELISA (e.g., GDF15 and corticosterone). The pelleted blood cells were diluted 1:1 in PBS. PBMCs were isolated by Ficoll‐Paque PLUS (GE Healthcare) density gradient centrifugation (1500 × g, 30 min, 20°C). Cells at the plasma‐Ficoll interface were collected, washed twice with PBS, resuspended in TRIgent (Mei5Bio, #MF034‐01), and stored at −80°C prior to RNA extraction and qPCR or transcriptome sequencing.

Brains were rapidly extracted and either snap‐frozen in liquid nitrogen for molecular analyses or fixed in 4% paraformaldehyde for histology.

### 4.4. GDF15‐KO Knockout Mice

GDF15‐KO knockout mice were purchased from Cyagen (Strain S‐KO‐07013). All the mice were maintained at less than 5 per cage in an SPF facility, and were allowed free access to food. The CRS and euthanasia, and tissue collection protocol were the same as ahead.

### 4.5. Immunofluorescence Staining

Coronal sections (20 μm) from 4% PFA‐fixed brains underwent antigen retrieval in citrate buffer (pH 6.0, 95°C, 20 min). After blocking with 5% bovine serum albumin + 0.3% Triton X‐100 (1 h, RT), sections were incubated overnight at 4°C with Iba1 (Servicebio, #GB12105‐100), and GDF15 (Proteintech, #27455‐1‐AP). After TBS‐T washes, species‐matched secondaries were applied: Alexa Fluor 594 donkey anti‐rabbit (Invitrogen #A‐21207) and DAPI to counterstain nuclei (1 μg/mL, 5 min). Slides were scanned with Panoramic MIDI.

### 4.6. Nissl Staining

Mice brains were postfixed over 24 h, dehydrated in 30% sucrose, and embedded in paraffin. Coronal sections (20 μm) encompassing the hippocampus were cut using a rotary microtome (Leica RM2235). Sections were stained with filtered 0.1% Cresyl Violet (Sigma, #C5042) at 50°C for 10 min, differentiated in 95% ethanol/0.1% acetic acid for 30 s, and dehydrated in absolute ethanol. Slides were scanned with Panoramic MIDI.

### 4.7. Golgi Staining

The entire brains were immersed in freshly prepared Golgi solution (Servicebio, #G1069‐20). The brains were stored in the dark at room temperature for 14 days, with the solution changed once after the first 48 h. Subsequently, the brains were transferred to a cryoprotectant solution (e.g., 30% sucrose) and stored at 4°C in the dark for at least 72 h before sectioning. Coronal brain sections (150 μm thickness) containing the prefrontal cortex and hippocampus were obtained using a vibratome (e.g., Leica VT1000S). The sections were mounted onto gelatin‐coated microscope slides, allowed to dry naturally in the dark, and then processed according to the kit instructions. The staining process involved sequential rinsing in double‐distilled water, development in a developer solution, dehydration through a graded ethanol series, clearance in xylene, and finally coverslipping with a nonaqueous mounting medium.

### 4.8. Transcriptome Sequencing

Total RNA was extracted from PBMC using TRIgent (Mei5Bio, #MF034‐01), with integrity verified (RIN >8.0, Agilent 2100). RNA sequencing was performed by Annoroad Gene Technology (Beijing, China) on the Illumina NovaSeq 6000 platform.

### 4.9. ELISA

Plasma GDF15 and corticosterone levels were quantified using a commercial Mouse GDF15 ELISA Kit (Elabscience, #E‐EL‐M0604) and QuicKey Pro Mouse CORT (Corticosterone) ELISA Kit (Elabscience, #E‐OSEL‐M0001). All assays were performed in duplicate strictly according to the manufacturer’s protocol. The absorbance was measured at 450 nm with a wavelength correction set to 570 nm using a microplate reader. The concentrations of GDF15 and corticosterone were interpolated from the standard curve and expressed as picograms per milliliter (pg/mL) for serum.

### 4.10. Cell Experiment

BV‐2 cells were maintained in DMEM high glucose (Gibco, #11965092) supplemented with 10% heat‐inactivated FBS (Kang Yuan Biology, #KY‐01002) and 1% penicillin‐streptomycin (Servicebio, #G4003‐100 ML) at 37°C/5% CO_2_. Cells were passaged at 80% confluence using 0.25% trypsin‐EDTA (3 min at 37°C) and seeded at 5 × 10^4^ cells/cm^2^. For cryopreservation, cells were resuspended in 90% FBS/10% DMSO at 2 × 10^6^ cells/mL and frozen at −1°C/min. Thawed cells were passaged once before experiments to ensure phenotype stability. BV‐2 cells were seeded in 12‐well plates (Corning, #3513) at 4 × 105 cells/well 24 h before treatment. At 70%–80% confluence, cells were washed with PBS and serum‐starved in DMEM (Gibco, #11965092) for 1 h. Recombinant mouse GDF15 protein (100 ng/mL) and Selumetinib (Selleck, #S1008, 10 μm) were added for 30 min, followed by stimulation with LPS (Sigma, #L2880; 500 ng/mL) for 6 h. Treated BV‐2 cells were washed twice with ice‐cold PBS and processed in parallel for protein or RNA analysis.

### 4.11. shRNA Knockdown in BV‐2 Cells

BV2 cells were seeded at 70% confluence in six‐well plates. The following day, cells were transduced with the GDF15‐in‐pLKO.1‐EGFP‐puro plasmid (GENERAL BIOL, #G0360126‐1) with lentiviral particles. After 24 h, the medium was replaced with fresh complete growth medium. Forty‐eight hours post‐transduction, cells were selected with 3 μg/mL puromycin (Solarbio, #P8230) for 7 days. Surviving polyclonal populations were expanded and maintained in medium containing 1 μg/mL puromycin. Western blotting was used to test the knockdown efficiency. The successfully knocked‐down cells were used for subsequent experiments.

### 4.12. RT‐qPCR

Total RNA was extracted using TRIgent (Mei5Bio, #MF034‐01). cDNA was synthesized from 1 μg RNA using Hifair II 1st Strand cDNA Synthesis SuperMix for qPCR (gDNA digester plus) (Yeasen, #11123ES60). qPCR reactions were performed in triplicate with SYBR Green Pro Taq HS qPCR Kit (Accurate Biology, #AG11701) on the Roche LightCycler 480 II system under the following conditions: 95°C/30 s; 40 cycles of 95°C/5 s, 60°C/30 s. Melt curve analysis (65–95°C) confirmed primer specificity. Data were analyzed by the 2^-ΔΔCt^ method. The primer sequences are shown below (5^′^–3^′^) in Table 1.

**Table 1:** Sequences of primers used in RT‐qPCR.

Gene	Forward primer	Reverse primer
*Il-1*β	GCAACTGTTCCTGAACTCAACT	ATCTTTTGGGGTCCGTCAACT
*Il-6*	TAGTCCTTCCTACCCCAATTTCC	TTGGTCCTTAGCCACTCCTTC
*Il-10*	CTTACTGACTGGCATGAGGATCA	GCAGCTCTAGGAGCATGTGG
*Tnf-α*	CCCTCACACTCAGATCATCTTCT	GCTACGACGTGGGCTACAG
*Tgf-β*	CCACCTGCAAGACCATCGAC	CTGGCGAGCCTTAGTTTGGAC
*GDF15*	CTGGCAATGCCTGAACAACG	GGTCGGGACTTGGTTCTGAG
*β-actin*	GGCTGTATTCCCCTCCATCG	CCAGTTGGTAACAATGCCATGT

### 4.13. Western Blotting

Mice tissue or BV‐2 cells were lysed in RIPA buffer (50 mM Tris‐HCl pH 7.4, 150 mM NaCl, 1% NP‐40, 0.5% sodium deoxycholate, 0.1% SDS) supplemented with 1× protease inhibitor cocktail (Roche, #04693159001) and 1 mM PMSF. Lysates were centrifuged at 12,000 × g for 15 min at 4°C. Supernatant protein concentrations were determined by BCA assay with absorbance measured at 562 nm. Equal amounts of protein (20 μg/lane) were separated on 10% SDS‐PAGE gels (120 V, 90 min) and transferred to PVDF membranes (Millipore, #IPFL00010) at 300 mA for 1.5 h. Membranes were blocked with 5% nonfat milk in TBST (Tris‐buffered saline + 0.1% Tween‐20) for 1 h at RT, then incubated overnight at 4°C with primary antibodies: (1) Phospho‐p44/42 MAPK Rabbit mAb (1:2000, Cell Signaling Technology, #4370). (2) p44/42 MAPK (Erk1/2) Rabbit mAb (1:2000, Cell Signaling Technology, #4695). (3) β‐Tubulin Antibody (1:5000, Cell Signaling Technology, #2146). (4) GDF15: Anti‐GDF15 antibody (1:5000, Abcam, #ab105738). (5) GAPDH: Anti‐GAPDH HRP conjugated antibody (1:5000, BOSTER, #A00227‐HRP). After TBST washes (3 × 10 min), membranes were incubated with HRP‐conjugated secondary antibodies: (1) goat anti‐rabbit (1:5000, CST, #7074S) for p‐ERK1/2, ERK1/2, and GDF15. (2) goat anti‐mouse (1:5000, CST, #7076S) for β‐Tubulin (1 h, RT).

### 4.14. Statistical Analysis

Outliers were identified using the ROUT method (*Q* = 1%). Data are presented as mean ± SD. All statistical analyses were performed using GraphPad Prism 10.0, with statistical significance defined as *p*  < 0.05. Normality was assessed by the Shapiro–Wilk test (*α* = 0.05). Homogeneity of variance was confirmed with Levene’s test. Based on these assessments: For normally distributed data with equal variance: Multigroup comparisons used one‐way ANOVA followed by Dunnett’s post hoc test. For normally distributed data with unequal variance: Multigroup comparisons used Welch’s ANOVA followed by Dunnett’s T3 post hoc test. For nonnormally distributed data: Multigroup comparisons used Kruskal–Wallis test followed by Dunn’s post hoc test.

## Funding

This work was supported by the National Natural Sciences Foundation of China (Grants 82171753 and 82371776) and the Beijing Natural Science Foundation (Grant 7244377).

## Conflicts of Interest

The authors declare no conflicts of interest.

## Supporting Information

Additional supporting information can be found online in the Supporting Information section.

## Supporting information


**Supporting Information** Figure S1 presents the comparison between GDF15 knockout mice and wild‐type mice after they underwent the same chronic restraint stress procedure. This included behavioral tests, detection of inflammatory factors, and the activation of the ERK signaling pathway in the hippocampus tissue. The results showed that GDF15 knockout led to an increase in resistance to chronic restraint stress.

## Data Availability

The data that support the findings of this study are available from the corresponding author upon reasonable request.
