# Supplementary material for: Exercise Attenuates Neuroinflammation in Chronic Restraint Stress Induced Depression Model by Downregulating GDF15 Expression
Source: J Immunol Res. 2026 Apr 9;2026:6816624. doi: 10.1155/jimr/6816624 (PMC13581054; doi:10.1155/jimr/6816624)
Supplement: Supplementary file 1 [file JIMR-2026-6816624-s001.pdf]

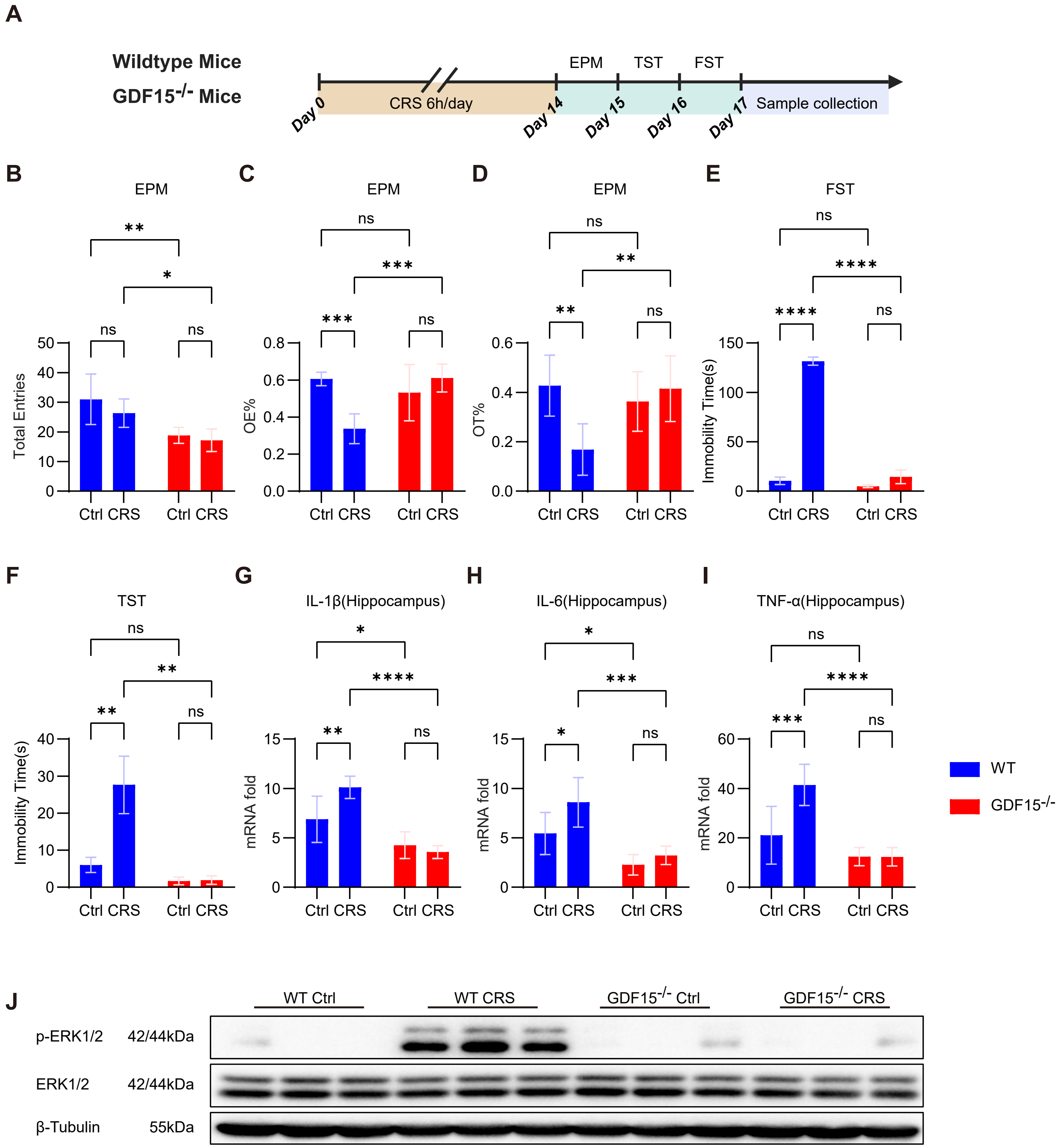
A
Wildtype Mice
GDF15-/- Mice
EPM
TST
FST
CRS 6h/day
Sample collection
Day 0
Day 14
Day 15
Day 16
Day 17
B
EPM
Total Entries
C
EPM
OE%
D
EPM
OT%
E
FST
Immobility Time(s)
F
TST
Immobility Time(s)
G
IL-1β(Hippocampus)
mRNA fold
H
IL-6(Hippocampus)
mRNA fold
I
TNF-α(Hippocampus)
mRNA fold
Ctrl CRS
Ctrl CRS
WT
GDF15-/-
J
WT Ctrl
WT CRS
GDF15-/- Ctrl
GDF15-/- CRS
p-ERK1/2 42/44kDa
ERK1/2 42/44kDa
β-Tubulin 55kDa
